# 3D CT volumetric analysis of spinal canal volume in Bichon Frisé, Dachshund, and French Bulldog: correlation with neurologic severity in thoracolumbar disc herniation

**DOI:** 10.3389/fvets.2026.1766999

**Published:** 2026-02-06

**Authors:** Ionuț Claudiu Voiculeț, Robert Cristian Purdoiu, Radu Lăcătuș, Felix Daniel Lucaci, Teodora Sonia Patrichi, István Farkas, Florin Ioan Beteg, George Tudor, Sorin Marian Mârza

**Affiliations:** 1 Clinical Science Department, Faculty of Veterinary Medicine, University of Agricultural Sciences and Veterinary Medicine, Cluj-Napoca, Romania; 2 Assisi Veterinary Clinic, Dublin, Ireland

**Keywords:** 3D segmentation, breed predisposition, canine intervertebral disc disease, comparative imaging, spinal canal stenosis

## Abstract

**Introduction:**

The intervertebral disc herniation (IVDH) is one of the most common causes of spinal injury in dogs. Certain breeds, notably chondrodystrophic dogs, are predisposed to severe thoracolumbar IVDH due to anatomical and genetic factors.

**Methods:**

This study applied three dimensional (3D) volumetric imaging analysis to compare the spinal canal volume and to investigate the relationship between canal volume and neurological symptoms severity. We retrospectively analyzed 21 dogs (7 Bichon Frisé, 7 Dachshund and 7 French Bulldogs) with imaging confirmed thoracolumbar disc herniation. Computed tomography (CT) scans were segmented using 3D Slicer to quantify the bony spinal canal volume from the cranial to caudal end of the vertebral column.

**Results and discussions:**

French Bulldogs showed a significantly larger mean spinal canal volume than those of Dachshunds and Bichons (*p* < 0.05 for both comparisons). Across all dogs, smaller canal volumes were associated with more severe neurological deficits (Spearman *ρ* ≈ −0.72, *p* = 0.0014). Across all breeds, disc herniations were most frequently observed at the thoracolumbar junction (T12–L2; 5 of 21 cases), but substantial overlap existed with neighboring segments. The thoracolumbar junction is especially susceptible to disc herniation because it is a transition zone between the rigid thoracic spine and the mobile lumbar spine, where mechanical stress is concentrated and spinal canal reserve space is limited. The 3D volumetric analysis of the vertebral canal is a feasible adjunct to routine imaging and reveals significant breed specific differences. Narrower canal volume appears to exacerbate the impact of disc herniation on the spinal cord, suggesting that canal size is a contributing risk factor for neurologic severity. A critical volume threshold (~1,700 mm^3^ per vertebral segment) was observed, under which dogs were markedly more likely to develop severe deficits.

## 1 Introduction

Neurologic impairment due to intervertebral disc herniation (IVDH) is frequently encountered in small animal practice and is a leading cause of canine spinal disorders (1). Certain dog breeds are particularly predisposed, owing to inherited chondrodystrophy and distinctive vertebral column conformations. For example, Dachshunds have an approximately 15–20% lifetime prevalence of IVDH and can represent up to 45–62% of all disc herniation cases in clinical surveys (2–4). Other small breeds, such as the Bichon Frisé (and Maltese) and the French Bulldog, are also widely recognized for increased risk of thoracolumbar IVDH (5, 6). This breed predisposition has a genetic basis: a fibroblast growth factor 4 (FGF4) retrogene insertion on canine chromosome 12 causes chondrodystrophic dwarfism and greatly heightens the probability of intervertebral disc degeneration in affected dogs (7). French Bulldogs, for instance, often carry this FGF4 retrogene and consequently exhibit both early disc degeneration and a high incidence of congenital vertebral malformations (e.g., hemivertebrae) (8, 9). These malformations can lead to spinal canal deformities or instability that compound the risk of spinal cord compression in the event of a disc extrusion (10). Therefore, breed specific anatomical factors such as a relatively narrow spinal canal in Dachshunds or vertebral anomalies in French Bulldogs are thought to modulate the severity of neurologic injury during IVDH events (2, 11).

Advanced diagnostic imaging is integral to the evaluation of canine IVDH (1, 12, 13), and magnetic resonance imaging (MRI) is considered the gold standard for identifying disc extrusions and assessing spinal cord compression or parenchymal changes. However, computed tomography (CT) is more widely accessible in many veterinary centers and can clearly depict osseous structures and mineralized disc material. CT myelography or plain CT is often used to confirm disc herniations when MRI is unavailable (1). Beyond simply localizing disc extrusions, imaging can yield quantitative morphological data that may have prognostic significance. Prior studies have demonstrated that linear measurements of the vertebral canal and body on CT and low field MRI are highly repeatable and in good agreement between modalities (1, 3, 12). This suggests that objective morphometric parameters of the spine can be measured reliably in clinical imaging. Indeed, vertebral canal diameter or crossectional area has been correlated with certain neurologic conditions (e.g., wobblers syndrome) in dogs (3, 14), and in humans, canal stenosis is a well-known factor influencing outcomes in disc herniation and myelopathy (15). However, to date there is limited information on the volumetric capacity of the canine spinal canal in different breeds and how this may relate to disc herniation severity. Most veterinary studies focus on the degree of spinal cord compression visible on MRI (such as percent canal compromise or cord signal changes) as predictors of outcome (16), rather than intrinsic canal size. We theorized that breeds with smaller spinal canal volume (relative to spinal cord size) have less “reserve space” for cord displacement during an acute disc extrusion, resulting in more severe neurologic injury for a given disc herniation volume.

In this context, we set out to integrate 3D volumetric analysis into the evaluation of canine IVDH.

The objectives of the study were: (1) to quantify the total spinal canal volume in three dog breeds predisposed to disc herniation (Bichon Frisé, Dachshund, French Bulldog) using retrospective CT imaging data; (2) to compare these volumetric measurements between breeds and determine if significant morphological differences exist; (3) to investigate the relationship between spinal canal volume and the severity of neurological deficits at presentation; and (4) to explore a potential critical volume threshold beyond which the risk of severe neurologic injury increases. By improving our understanding of breed associated vertebral canal variation, this research aims to enhance prognostic precision and inform clinical decision making (e.g., urgency of surgery) in dogs suffering from disc herniation. Our *a priori* goal was to test whether, among dogs already showing intervertebral disc disease (IVDD), smaller bony canal volume is associated with poorer neurological grade and whether breeds differ in canal capacity. These three breeds were selected because they are all predisposed to thoracolumbar intervertebral disc disease, yet they exhibit distinct body conformations and spinal proportions. Dachshunds represent a long-bodied chondrodystrophic phenotype with relatively narrow canals, French Bulldogs a short, compact brachycephalic conformation with broader vertebral dimensions, and Bichon Frisés an intermediate small-breed morphology. Comparing these groups therefore allowed assessment of how canal volume relates to neurological severity across different spinal shapes within a shared disease predisposition.

## 2 Materials and methods

### 2.1 Case selection and inclusion criteria

This study was conducted as a retrospective observational analysis of clinical cases. Medical records from the Veterinary Teaching Hospital of Faculty of Veterinary Medicine Cluj Napoca, Romania were reviewed to identify dogs that met the inclusion criteria: (a) Breed – the dog was a Bichon Frisé (including Maltese Bichon), Dachshund, or French Bulldog (breeds selected based on known IVDH predisposition) (2, 5, 17); (b) Diagnosis – an intervertebral disc herniation in the thoracolumbar spine (T12–L2 region) confirmed by advanced imaging (in these breeds this was typically a Hansen type I disc extrusion); and (c) Imaging Data – availability of a DICOM dataset images suitable for high resolution volumetric imaging study of the spine sufficient to allow 3D analysis of the bony spinal canal.

We retrospectively assigned each case a clinical neurologic severity grade on a simple three tier scale. Mild (Score 1): spinal pain only, no detectable motor or proprioceptive deficits – the dog is ambulatory with normal deep pain sensation. Moderate (Score 2): paraparesis (ambulatory or non-ambulatory) with intact deep pain. Severe (Score 3): paraplegia and/or loss of deep pain sensation. Scores were assigned from the presentation neurologic examination; borderline cases were conservatively assigned the higher severity. Scoring was performed without knowledge of canal volume measurements and treated as ordinal for analysis.

We initially identified 21 dogs (7 of each breed) that fulfilled these criteria and were examined between March 2022 and January 2024. All dogs had clinical signs consistent with thoracolumbar IVDD (such as back pain, ataxia or paresis, with or without paralysis) and underwent diagnostic CT scanning. Cases with a history of major spinal trauma, neoplasia, or other spinal pathology (including non-compressive Hansen type III disc extrusions) were excluded to avoid confounding factors. We also excluded any dog that did not have a complete imaging series or had excessive image artifacts preventing segmentation. Owner consent for diagnostic procedures was obtained in all instances as per hospital protocol, and the use of these data for research was approved by the institutional clinical review board (490/21.01.2025).

### 2.2 Imaging protocol and 3D segmentation

All dogs were imaged using a multislice helical CT scanner (Siemens Somatom Scope 16-slice, Siemens Healthineer, Germany, Europe). General anesthesia or sedation was utilized to ensure the dogs remained motionless during image acquisition. Patients were positioned in dorsal recumbency with forelimbs extended, which aids in minimizing spinal column rotation artifacts. The scan range encompassed the entire vertebral column from the first cervical vertebra (C1) to the sacrum (S1-S3). Scan parameters were consistent across cases: tube voltage 120 kVp, tube current 200–250 mA, slice thickness 1.5 mm, pitch ~0.8, and a high resolution bone reconstruction algorithm with multiplanar reconstructions in sagittal, transverse, and coronal planes. No intravenous contrast medium was used, as the primary goal was bony anatomy visualization; however, in some instances a attenuation effect was achieved by the naturally present epidural fat and contrast differences, allowing the spinal cord and subarachnoid space to be distinguished within the canal.

Images were viewed using both a standard bone window (level ~300 HU, width ~1,500 HU) and a soft tissue window (level ~50 HU, width ~350 HU) to optimize discrimination of osseous and soft tissue structures.

All scans were initially reviewed by an experienced veterinary radiologist for diagnostic purposes. No additional blinded reevaluation of the CT images was performed for this study – the analyses were based on the clinical reads and volumetric segmentation. This could introduce some bias, although the canal volume measurement itself is an objective process.

**Figure 1:**
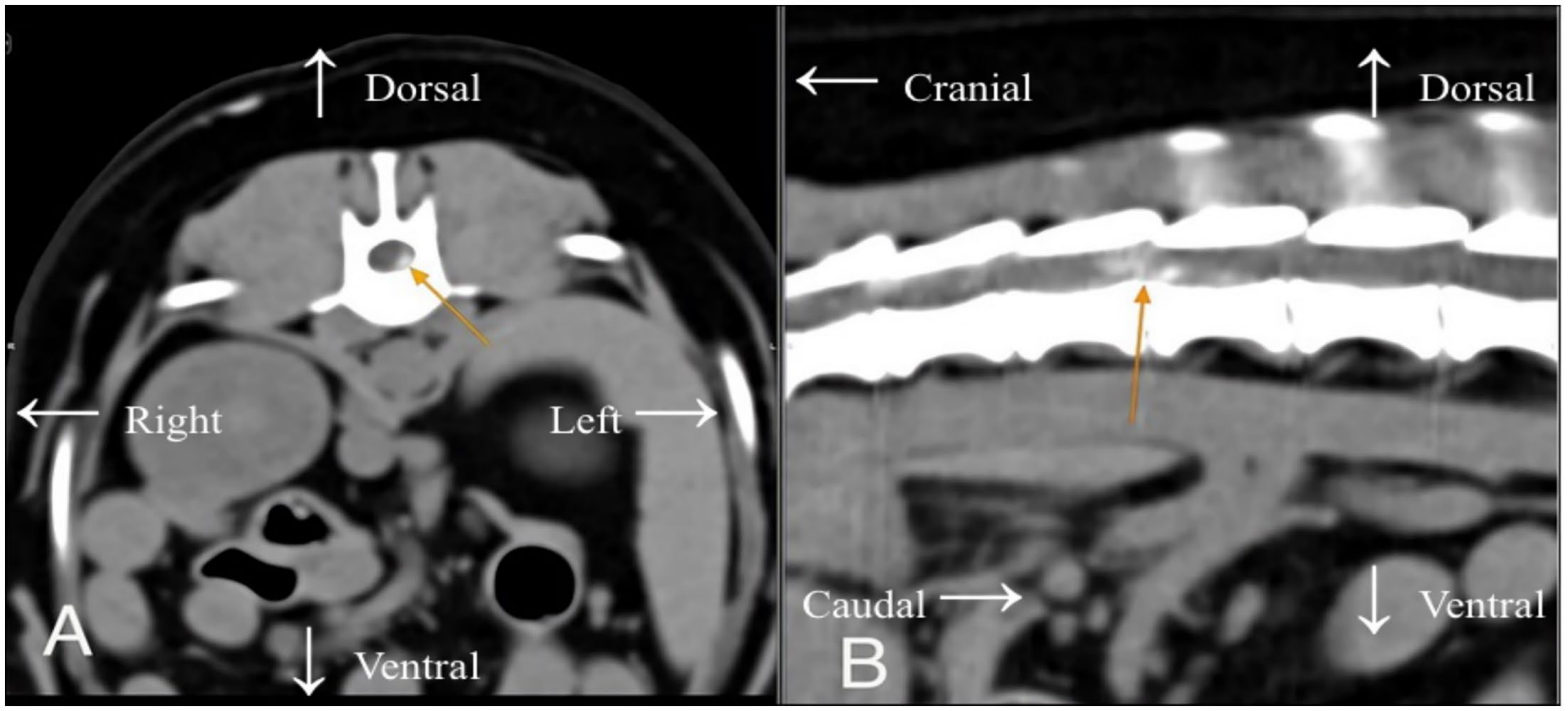
Bichon Frisé case – **(A)** axial view and **(B)** sagittal view: CT image at T13–L1 showing a left sided calcified disc extrusion (orange arrow) compressing the spinal cord.

**Figure 2:**
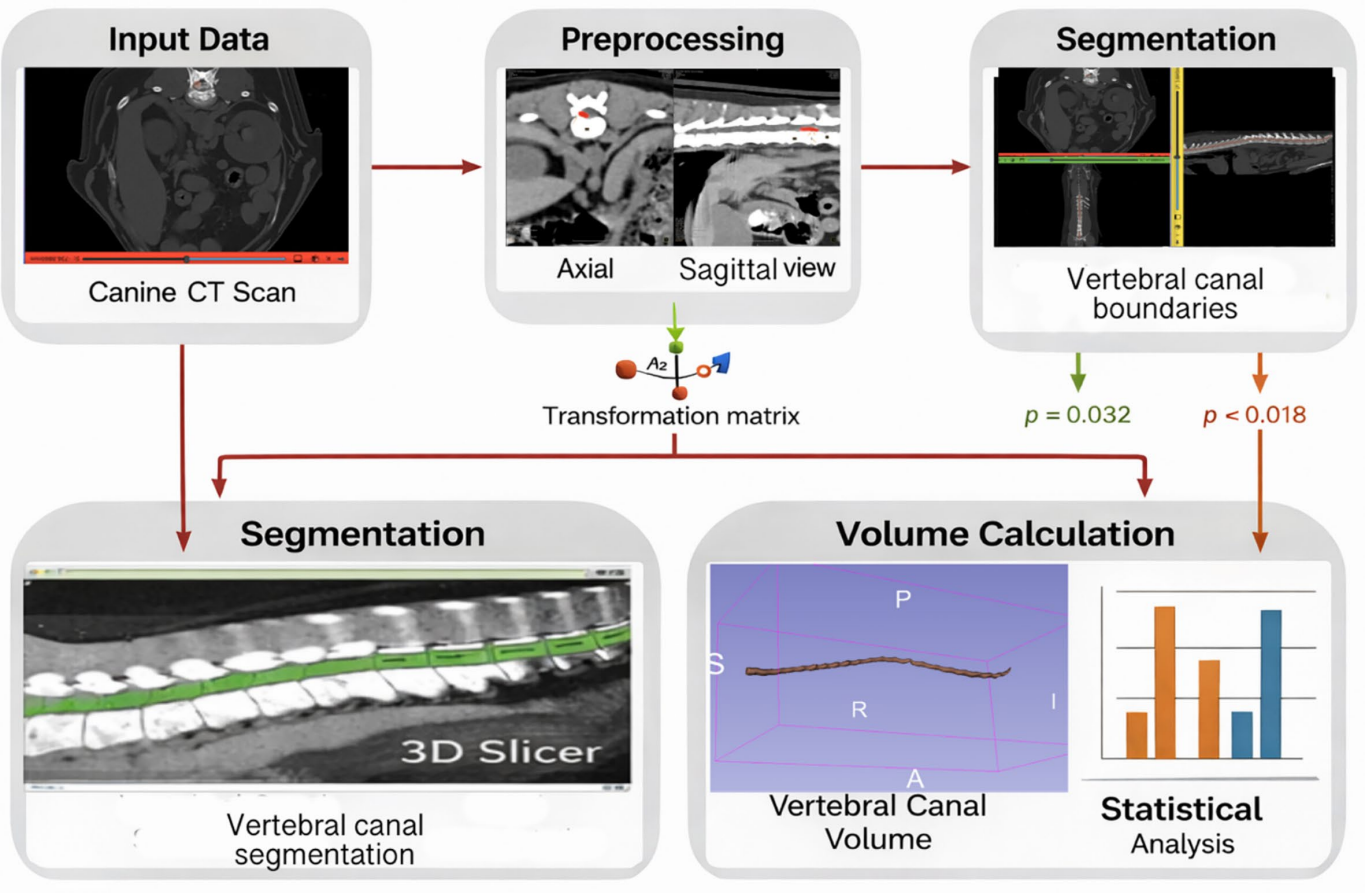
Visual summary of the image processing and segmentation pipeline used to quantify vertebral canal volume from thoracolumbar CT scans, including vertebral level identification, three-dimensional segmentation of the bony canal, and volumetric measurement.

**Figure 3:**
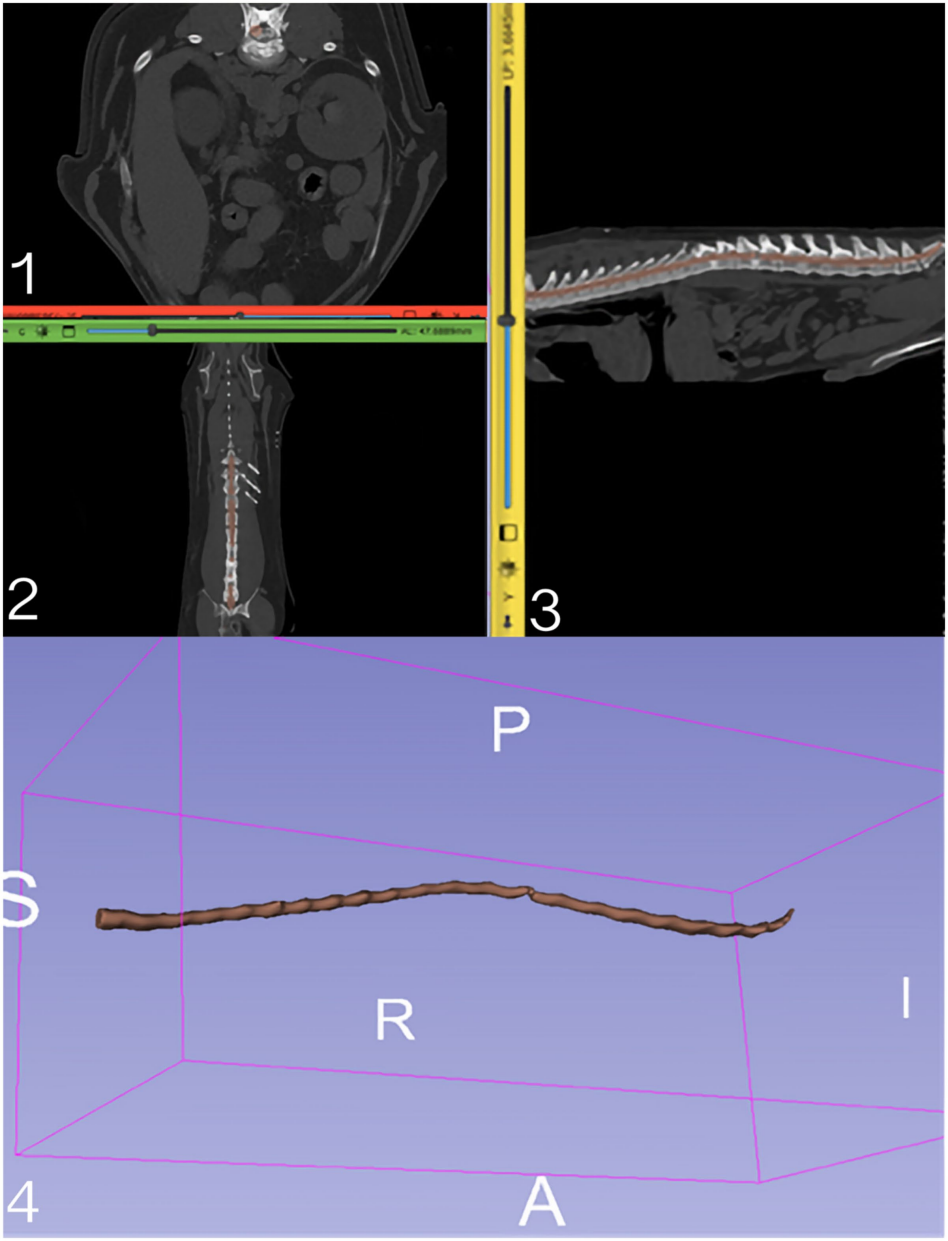
Illustrate **1–3**: 3D Slicer axial, dorsal, and sagittal multiplanar reconstruction of the scanned area and mask applied for the spinal cord; **4**: 3D reconstruction of the Bichon’s spinal canal (purple) demonstrates an overall volume and severe localized narrowing at the extrusion site. Legend for annotation in picture **4**: S, superior (cranial); I, inferior (caudal); A, anterior (ventral); P, posterior (dorsal); R, right; L, left.

The CT data for each dog were exported in DICOM format and imported into 3D Slicer (v5.6.0 and v5.8.1, open source software, Cambridge, MA, USA) for volumetric analysis. Within 3D Slicer, the Segment Editor module was used to create a 3D segmentation of the vertebral canal (i.e., the osseous spinal canal through which the spinal cord passes). The segmentation process involved an initial threshold based selection of the canal space, taking advantage of the clear density difference between bone (vertebral arch) and the canal lumen on CT. An intensity threshold was applied to highlight voxels corresponding to the canal (which, being filled with cerebrospinal fluid and soft tissue, appear hypoattenuated relative to bone). A localized thresholding (region-growing) approach was then used to refine the selection specifically within each vertebral segment. The segmented region was further edited manually as needed: the paint and erase tools were used in axial, sagittal, and coronal views to include any regions of the canal that the automated step missed, or to exclude any partial volume artifacts or foramina that were erroneously included. Care was taken to ensure that the entire bony spinal canal from C1 to L7 (S1 if included) was segmented for each dog, yielding a total spinal canal volume per subject (later expressed per vertebra for threshold analyses). In two French Bulldog cases with vertebral malformations, particular attention was paid to correctly delineate the canal in those deformed segments. After achieving a satisfactory segmentation (validated by inspecting the 3D model and multiplanar images to confirm the canal was fully and exclusively captured), the Segment Statistics module was used to calculate the total volume of the segmented canal. The software computes volume by multiplying the number of voxels in the segment by the volume per voxel (based on slice thickness and in plane pixel size). The resulting spinal canal volume for each dog was recorded in cubic millimeters (mm^3^) (Figures 1–3).

We also noted each dog’s affected disc site (the vertebral level of the herniation as seen on CT) and whether any obvious canal stenosis or concurrent malformations were present at other levels.

### 2.3 Data analysis and statistics

All measured and collected variables were compiled, including breed, age, sex, spinal canal volume, and clinical severity score for each dog. Summary statistics were generated for each breed group. We calculated the mean canal volume, standard deviation (SD), and coefficient of variation (CV = SD/mean × 100%) within each breed. The CV provides a dimensionless measure of relative variability; a low CV indicates the individuals of that breed had fairly homogeneous canal sizes.

Given the small sample size (*n* = 7 per breed) and a Shapiro–Wilk test indicating non-normal distributions, we used a non-parametric Kruskal–Wallis test (*α* = 0.05) to compare canal volumes among the three breeds. If an overall difference was detected, pairwise comparisons were performed with Dunn’s test (Bonferroni-adjusted) to determine which breeds differed.

We also assessed the association between canal volume and neurologic grade using Spearman’s rank correlation. Additionally, a binary logistic regression (outcome: severe deficit vs. non-severe) was performed and a receiver operating characteristic (ROC) curve was generated to identify a potential canal volume cutoff associated with severe neurologic impairment. This analysis was performed to find an approximate critical canal volume below which the likelihood of severe neurologic impairment markedly increases.

All statistical analyses were conducted using Prism 9 (GraphPad Software, San Diego, CA, USA) and SPSS (IBM Corp., Armonk, NY, USA). A *p* value < 0.05 was considered statistically significant for all tests.

## 3 Results

### 3.1 Study population

A total of 21 dogs (7 Bichon Frisés, 7 Dachshunds, and 7 French Bulldogs) met the inclusion criteria. The sex distribution comprised 11 males and 10 females overall, with both sexes represented in each breed group (Bichon: 5 males/2 females; Dachshund: 5 males/2 females; French Bulldog: 6 males/1 female). The median age was 6 years (range 3.5–10 years), with approximately 70% of dogs between 5 and 8 years of age. Neurological severity at presentation included eight dogs with severe deficits (Score 3), with the remaining dogs classified as moderate (Score 2) or mild (Score 1).

### 3.2 Breed differences in spinal canal volume

Descriptive statistics of spinal canal volume by breed are summarized in Table 1. The mean spinal canal volume was 14,117 ± 1,165 mm^3^ in French Bulldogs, 12,189 ± 821 mm^3^ in Dachshunds, and 12,971 ± 566 mm^3^ in Bichons. The coefficient of variation ranged from 5.4 to 9.4% across breeds.

**Table 1:** Spinal canal volume by breed.

Breed	*n* (dogs)	Mean volume (mm^3^)	SD (mm^3^)	CV (%)	Min (mm^3^)	Max (mm^3^)
Bichon Frisé (Maltese)	7	12,687.0	689.8	5.44	11,742.9	13,393.2
French Bulldog	7	14,116.9	1,165.0	8.25	12,734.8	15,713.2
Dachshund	7	12,711.8	1,199.7	9.44	11,624.9	14,874.2

Descriptive statistics for the spinal canal volume in each breed. n, refers to the number of dogs (with adequate imaging volume) in each breed; SD, standard deviation; CV, coefficient of variation; min, minimum; max, maximum observed value.

#### 3.2.1 Statistical comparison

**Figure 4:**
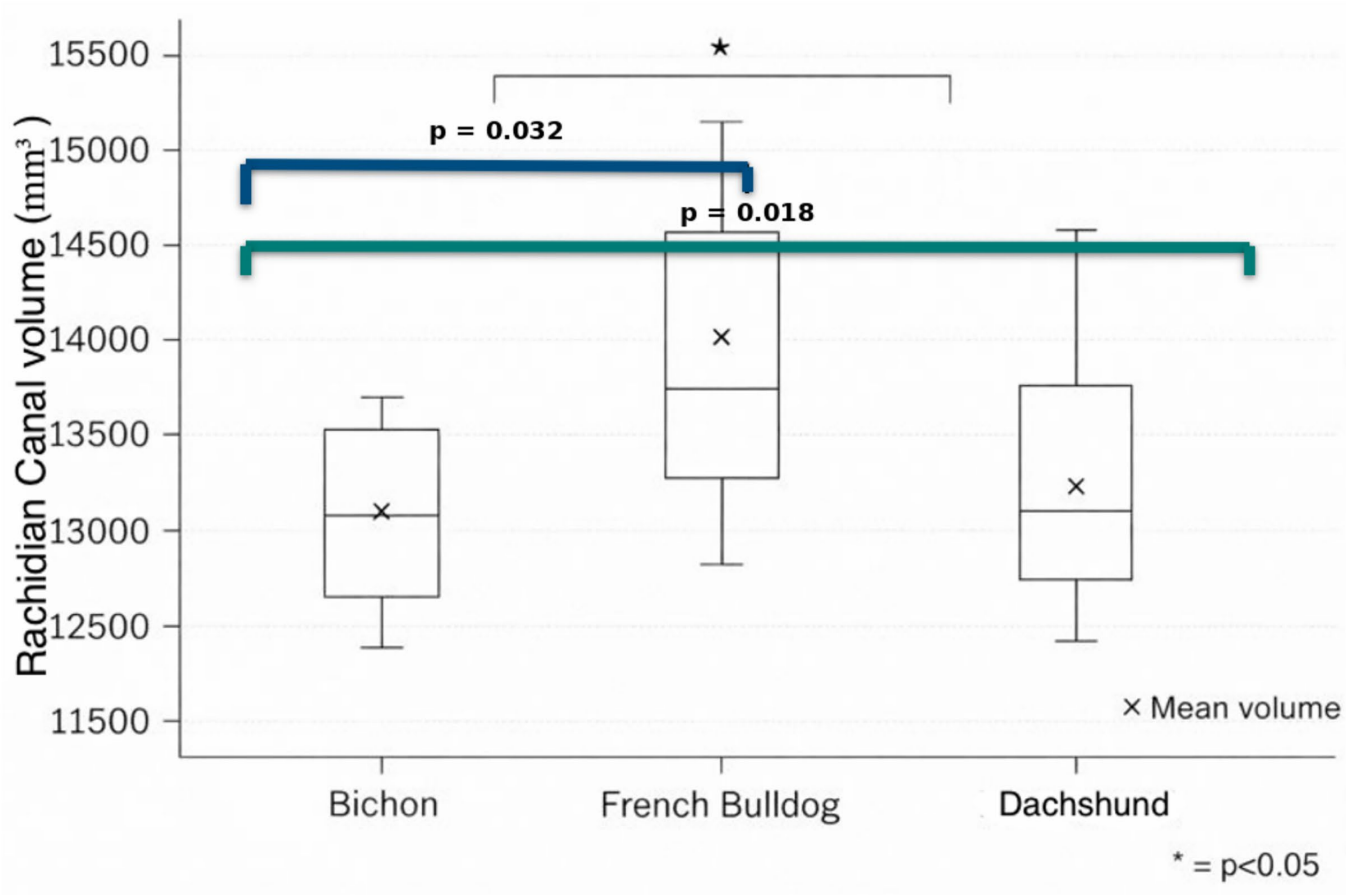
Illustrate the distribution of canal volumes by breed. The box’s central line indicates the median, and the “×” marker denotes the mean volume. French Bulldogs have both a higher median and a higher mean canal volume compared to Dachshunds and Bichons. The Dachshund group also exhibits a wider range of volumes, with one or two individuals having notably larger canal volumes – those outlier dogs in fact had only mild neurologic signs, consistent with the overall trend – whereas the Bichon volumes are tightly clustered around the mean. Pairwise differences between breeds were assessed using Kruskal–Wallis with Dunn’s *post hoc* test. Brackets were used to show statistical significant comparison between *p < 0.05.

The Kruskal–Wallis test revealed a significant difference in spinal canal volumes among the three breeds (*p* = 0.046), therefore, we rejected the null hypothesis of equal medians and proceeded with pairwise comparisons. Post-hoc pair-wise comparisons (Dunn’s test with Bonferroni correction) showed that French Bulldogs had significantly larger spinal canal volumes than both Bichons (adjusted *p* = 0.03) and Dachshunds (adjusted *p* = 0.02), whereas Bichon vs. Dachshund was not significant (adjusted *p* = 0.65). In other words, the French Bulldog’s spinal canal volume was on average ~1,400 mm^3^ greater than that of the other two breeds. No difference was detected between Bichon and Dachshund canals; their volume ranges overlapped considerably (Figure 4).

### 3.3 Spinal canal volume vs. neurologic severity

A significant negative correlation was observed between spinal canal volume and neurological severity (Spearman *ρ* = −0.72, *p* = 0.0014). Among Dachshunds and Bichon Frisés, the lowest canal volumes in each breed were recorded in dogs classified as Score 3, whereas dogs classified as Score 1 or 2 exhibited higher canal volumes. In Dachshunds, severe cases (Score 3) had volumes of 12,101 and 12,843 mm^3^, while mild cases (Score 1) had volumes of 13,105 and 13,278 mm^3^. In Bichon Frisés, the lowest volumes (11,954 and 12,228 mm^3^; both Score 2) were observed in moderately affected dogs, whereas all dogs with mild deficits (Score 1) had canal volumes ≥ 12,354 mm^3^. In French Bulldogs, all measured canal volumes exceeded 13,000 mm^3^, and these dogs were classified as either Score 1 or Score 2.

**Figure 5:**
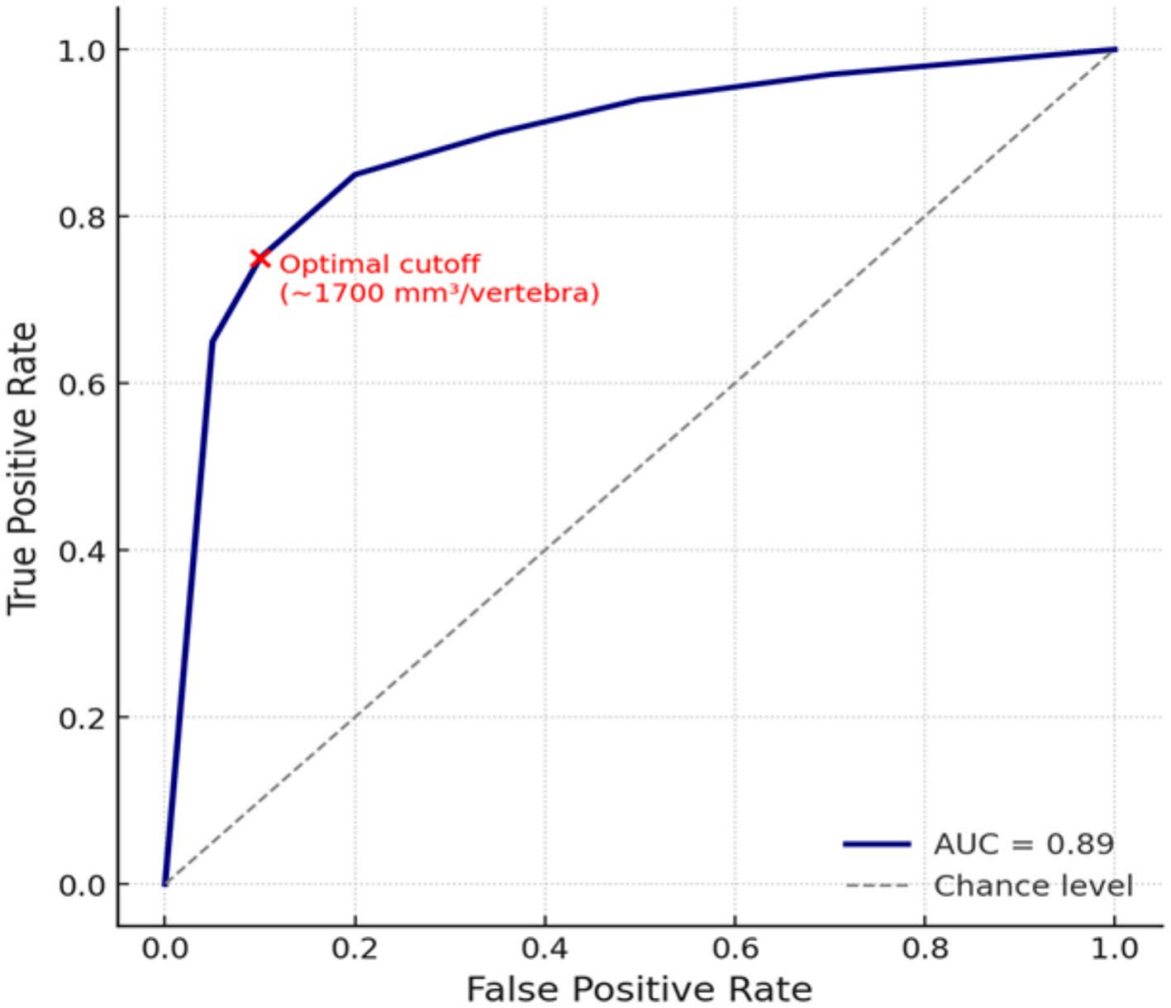
ROC curve for using spinal canal volume (per vertebra) to predict a severe neurologic outcome.

Logistic regression and ROC analysis supported a threshold effect. Figure 5 shows the ROC curve for using spinal canal volume to predict severe deficits: the area under the curve was ≈ 0.89, and an optimal cutoff of about 1,700 mm^3^ per vertebral segment (≈ 12,000 mm^3^ total across the thoracolumbar spine) distinguished dogs with severe neurologic injury (Score 3) from those with milder deficits (Score 1, Score 2) (sensitivity ~87%, specificity ~80%).

**Figure 6:**
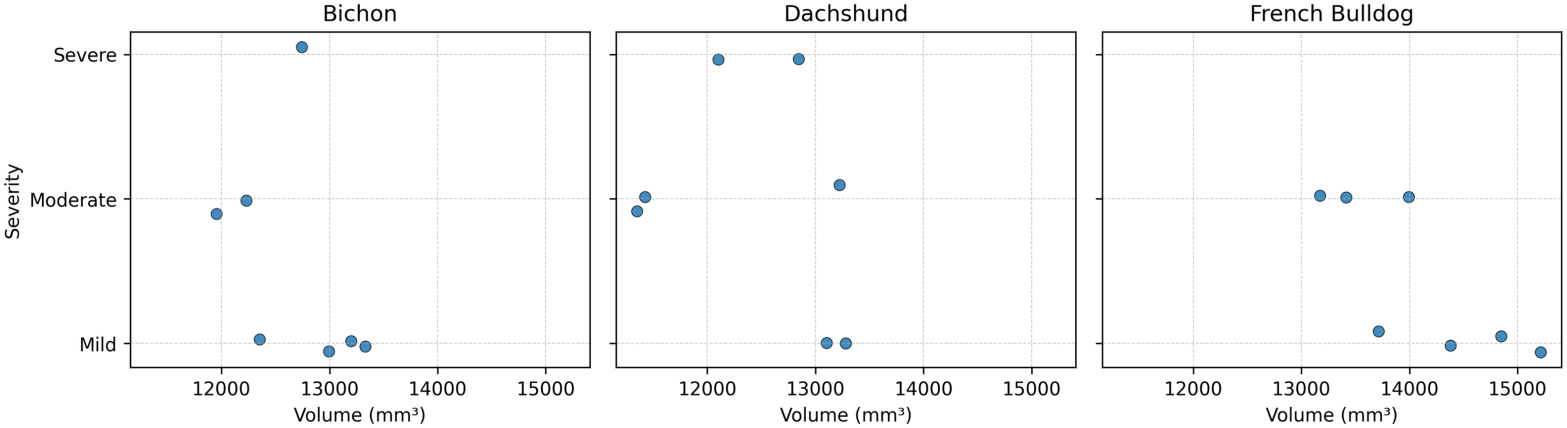
Scattered plot showing the cases distribution according to spinal canal volume and severity grade of compression.

These individual variations notwithstanding, the overall trend supports that canal volume is inversely proportional to neurologic injury severity, and a critical zone around 12,000 mm^3^ total volume (or ~1,700 mm^3^ per vertebra) exists below which dogs are much more likely to suffer profound deficits (Figure 6).

## 4 Discussion

In this study, we employed 3D volumetric analysis of spinal canal anatomy to investigate morphological differences among three dog breeds predisposed to intervertebral disc herniation, and to explore how these differences might affect clinical outcomes. To our knowledge, this is the first report to provide quantitative spinal canal volume data across these breeds and to correlate such data with neurologic severity in dogs with IVDH. The results demonstrate that French Bulldogs, Bichons, and Dachshunds – despite all being small breeds prone to disc disease – have distinguishable spinal canal morphologies, and that a smaller canal volume is associated with more severe neurologic injury from disc extrusion. The age distribution of the study population corresponds to the typical onset of degenerative intervertebral disc disease in predisposed breeds, which most commonly affects middle aged dogs (2). All dogs exhibited thoracolumbar neurological signs consistent with intervertebral disc herniation, and the index disc herniation in all cases was located within the T12–L2 region (2, 4, 6, 17).

### 4.1 Breed-specific findings

We found that French Bulldogs have, on average, a significantly larger spinal canal volume compared to Dachshunds and Bichon Frisés. This aligns with what one might expect from the skeletal build of these breeds. French Bulldogs are stout, short backed dogs; our finding suggests they possess a relatively spacious vertebral canal (perhaps as an adaptive trait or simply due to overall body dimensions). In contrast, Dachshunds – the classic chondrodystrophic breed – showed the smallest canal volumes. Dachshunds are genetically predisposed to chondroid degeneration of the intervertebral discs at a young age, owing in large part to the FGF4 retrogene insertion that defines chondrodystrophy (7). This genetic mutation not only causes premature disc calcification (10, 18), but is also associated with the dogs’ disproportionate dwarfism. Dachshunds’ narrow canals align with their conformation—long backs with short pedicles—yielding a smaller canal cross-section that magnifies the effect of any extrusion. Our data are concordant: Dachshunds both herniate more often and, with less reserve space, tend to present more severely. Bichon Frisé—though not classically chondrodystrophic—showed canal volumes similar to Dachshunds in this cohort, consistent with scaling effects in small dogs. MRI-based morphometric studies in dogs show that vertebral canal width and height scale with body weight, and that dogs of lower body mass exhibit higher spinal cord-to-canal ratios—reflecting reduced peri medullary reserve space—compared with heavier dogs within the same size category (1, 4, 19, 20); similarly, lower weight dogs have a higher spinal cord-to-canal width ratio, indicating less free space surrounding the cord (15, 19, 20).

Because vertebral canal volume is the product of both cross-sectional area and segment length, breed-related differences in spinal proportions (e.g., long, narrow canals in Dachshunds versus short, wide canals in French Bulldogs) may yield similar total volumes while conferring markedly different local biomechanical and neurological vulnerability at the level of disc herniation.

Accordingly, segment specific canal volume at the site of intervertebral disc herniation is likely a more biologically meaningful predictor of neurological severity than total canal volume alone, particularly in morphologically divergent breeds.

These breed differences have direct clinical implications. Dachshunds are notorious for severe IVDH – they comprised over 45% of the surgically treated disc cases in some studies (3, 5, 21), and about 20% of Dachshunds will experience disc herniation in their lifetime (5, 22, 23). Our data provide a structural rationale: a Dachshund’s spinal cord is at risk of being pinched in a confined space when a disc ruptures. Indeed, we observed Dachshunds often presented with high-grade injuries (paralysis), and even a relatively small disc extrusion can cause significant compression in them. French Bulldogs, on the other hand, showed a paradox: despite a larger canal (which should be protective), they can still suffer severe spinal issues. The discussion in the literature points to congenital vertebral malformations (like hemivertebrae, wedge vertebrae, and transitional vertebrae) being extremely common in French Bulldogs (8). These malformations can cause local kyphosis (angulation of the spine) and canal shape distortions. A French Bulldog might have a globally large canal volume, but a hemivertebra at (for example) T8 could significantly narrow the canal at that point and predispose to compression or instability at that site. Additionally, French Bulldogs often have multiple disc protrusions and a propensity for vertebral column instability (like “mini wobblers” syndrome). In our sample, no French Bulldog was deep pain negative – which might suggest that their generally larger canal gives some resilience, or it could be since French Bulldogs in our cohort were promptly treated or had more chronic courses. It’s known that French Bulldogs often present slightly younger for IVDD than Dachshunds and can have a high rate of needing surgery, often due to concurrent bony malformations (8). Our results underscore that canal volume is only one factor – in French Bulldogs, even though volume is ample, other factors (like vertebral anomalies or the presence of fibrous connective tissue proliferation around malformations) can lead to severe neurologic signs that are not volume dependent.

### 4.2 Volume vs. severity

The core finding of an inverse correlation between spinal canal volume and neurologic severity supports our hypothesis and agrees with clinical intuition. If the spinal canal is congenitally narrow (or “stenotic”), any extruded disc material will occupy a larger fraction of the available space, causing greater spinal cord deformation and compression. In human medicine, patients with developmentally narrow cervical canals are known to be at higher risk for acute cord injury from even minor disc bulges or trauma (the so called “functional spinal reserve” concept) (15). A similar concept likely applies in dogs – Dachshunds have virtually no reserve space in many cases, so a disc extrusion compresses the cord more severely than it might in a dog with a wide canal. Our data quantifies this relationship and even identifies a potential threshold (~1,700 mm^3^ per vertebra) below which the risk sharply rises. This per-vertebra value represents an average across the thoracolumbar region rather than a site specific measurement at the herniation level, as segmentation was performed for the entire bony canal. MRI-based morphometric studies of the canine thoracolumbar spine report mean vertebral canal cross-sectional areas of approximately 50–60 mm^2^ in dogs of moderate body mass and approximately 30–40 mm^2^ in lower weight dogs, reflecting a marked reduction in peri medullary reserve space in smaller dogs (15, 19, 20). It is intriguing to consider this threshold in anatomical terms: for a mid-back vertebra in a small dog, 1,700 mm^3^ roughly corresponds to a canal cross-sectional area of perhaps 50–60 mm^2^ (assuming an average vertebral length of ~30 mm). That cross-sectional area is quite small – likely barely enough to accommodate the canine spinal cord (which in a Dachshund may have a cross-sectional area of ~30–40 mm^2^) plus a thin layer of cerebrospinal fluid (CSF). So, a canal volume below 1,700 mm^3^/vertebra essentially means almost no extra space around the cord. This supports our observation that those dogs had effaced CSF on imaging and often severe deficits. This supports our observation that those dogs had effaced CSF on imaging and often severe deficits. Conversely, dogs above that threshold had some “buffer” space. These findings are also consistent with earlier qualitative observations: for example, a myelographic study noted that dogs with complete block of contrast flow (indicating no subarachnoid space visible) tended to have worse prognoses than dogs with remaining CSF space (16).

It is important to clarify that this threshold is not an absolute demarcation of outcome, but a point of significantly elevated risk. For instance, one Dachshund in our series had a canal volume around 11,700 mm^3^ and was paraplegic without deep pain (the most severe category), whereas a Bichon with a volume ~11,740 mm^3^ was paraplegic but still had pain (severe, but slightly better outcome). Conversely, a French Bulldog with ~12,800 mm^3^ volume was only paretic.

It should be noted that correlation does not prove causation – the relationship could also reflect that more severe disc extrusions cause secondary swelling that effectively “reduces” the functional canal space. However, in our measurements we captured the fixed bony canal diameter, which is a preexisting anatomical factor. In segments distant from the herniation site, the bony spinal canal remained morphologically normal, indicating that the volumetric differences observed primarily reflect intrinsic breed related anatomy rather than secondary remodeling caused by the acute disc extrusion. The consistency of an inverse correlation across this diverse sample (even with the French Bulldogs included, which had large canals and generally milder signs) suggests that intrinsic canal size is one contributing factor to outcome.

We also must acknowledge that neurologic severity is multifactorial. Spinal canal volume is one anatomical risk factor, but the volume of extruded disc material, the force/velocity of extrusion, and secondary hemorrhage or ischemia all critically influence outcome (16). Our case studies highlighted this: Bichon Frisé (Case 4, T13–L1) had a comparatively large spinal canal; however, a high velocity acute disc extrusion into this wide canal produced severe focal spinal cord compression and a concussive injury, resulting in severe neurological deficits. This is analogous to the acute non-compressive nucleus pulposus extrusion (ANNPE or Type III disc) phenomenon, where a small amount of disc material shot out at high speed can contuse the cord without compressing it, often leading to severe deficits despite no ongoing compression. In such cases, canal volume does not prevent injury because the mechanism is different (concussion rather than compression). In the Bichon’s surgery, we observed signs of myelomalacia (softening) on the cord surface, which aligns with a concussive injury. Fortunately, that dog recovered well, possibly due to timely surgery removing any remaining compression and allowing the cord to heal.

Another factor is duration of compression. A dog with a narrow canal might become paraplegic quickly, but with prompt surgery can recover if the cord is not irreversibly damaged. Conversely, a dog with a moderate canal might only be paretic but if left untreated for too long, chronic compression could cause progressive axonal loss. Our data being mostly acute presentations cannot directly speak to chronicity effects, but it is known that duration of compression negatively correlates with recovery in deep pain negative dogs (24, 25). Thus, canal volume is a piece of the puzzle for predicting outcome, best considered alongside other imaging features like intramedullary T2 hyperintensity on MRI (which indicates cord edema/ischemia and is a bad prognostic sign) (16). Future studies integrating volumetric canal measures with MRI findings could yield a more comprehensive prognostic model.

These CT-derived volumetric measurements of spinal canal capacity are consistent with previously published MRI morphometric reference ranges for normal canine spines; conventional MRI evaluations have established baseline spinal cord and canal dimensions and cord-to-canal ratios across thoracic and lumbar levels in neurologically normal dogs, and have shown that spinal canal size increases with body weight, providing an important comparative context for the volumetric differences observed in this study (26).

### 4.3 Clinical implications

Our findings align with previous imaging studies demonstrating that increased spinal cord compression at the site of intervertebral disc extrusion correlates with more severe neurological impairment in dogs with thoracolumbar IVDD, indicating that reduced functional reserve space and the magnitude of focal compression both contribute to poorer neurologic outcomes (27). The threshold of ~1,700 mm^3^ (which we translate to roughly “canal diameter less than X mm” in practice) could be used as a guideline: Dogs falling below that might be counseled as high risk. Indeed, we might suggest that Dachshunds with canal volume in the lowest quartile should have shorter intervals between onset and surgery for best outcomes. Our data even hint at specific recommendations: e.g., if a Dachshund’s CT shows canal volume < 12,000 mm^3^, and they are exhibiting serious neuro deficits, early surgery is recommended to avoid irreversible damage. On the other hand, in a French Bulldog with a large canal and only pain/ataxia, a more conservative approach might be justified, as observed in one French Bulldog case in our series (which did well without surgery). That said, one must be cautious: French Bulldogs can have hidden problems like hemivertebra – so if a French Bulldog has severe signs, one should investigate for malformations or concurrent issues, not be falsely reassured by a big canal measurement. In those dogs, advanced imaging (MRI in addition to CT) might be needed to identify lesions like spinal cord bruising or multiple compressive sites.

From a neurosurgical planning perspective, 3D models of the spinal canal (as we created) could be very useful. Segmental canal volume at the disc herniation level is therefore directly represented within the total thoracolumbar volume analyzed. They provide a patient specific map of where the canal might be unusually narrow or wide. Identification of dogs with reduced spinal canal reserve may have implications for risk stratification and clinical management; however, anatomical predisposition alone does not justify prophylactic surgery. Instead, such information may be used to guide monitoring strategies, owner counseling, activity modification, physiotherapy, and individualized medical or surgical decision making when clinical disease develops. The volumetric approach is an objective way to quantify stenosis, potentially more reproducible than subjective impressions on imaging. Incorporating such quantitative analysis into routine CT/MRI evaluation could refine our decision algorithms.

## 5 Limitations

Several limitations of this study should be acknowledged, many of which are inherent to its retrospective, clinically based design. First, the sample size was limited (*n* = 21 with volumetric data) and derived from a single referral center, which restricts statistical power and generalizability. Consequently, correlation analyses and the proposed volumetric threshold (e.g., ~1700 mm^3^) should be regarded as hypothesis generating rather than definitive, and larger multicenter cohorts will be required to refine such cutoffs.

A healthy control group was not included, as this was a retrospective study of dogs that underwent CT because of clinical thoracolumbar intervertebral disc herniation. Acquiring CT or MRI scans of neurologically normal dogs solely for research purposes is ethically and practically constrained, particularly in breeds not otherwise requiring imaging. Importantly, the objective of this study was not to define normative reference values, but to examine whether inter individual differences in vertebral canal volume among affected dogs relate to neurological severity. Thus, the absence of healthy controls does not invalidate the observed within cohort associations.

Similarly, the lack of MRI is not a limitation of the study design, as the aim was to investigate bony spinal canal geometry using CT, which remains the standard modality for assessing vertebral morphology and mineralized disc material in clinical IVDD. While MRI would be required to directly evaluate spinal cord edema, hemorrhage, or myelomalacia, these soft tissue changes are themselves influenced by the available canal space; therefore, a narrower bony canal may exacerbate the impact of swelling or hemorrhage even when these factors cannot be directly visualized on CT.

We did not exclude dogs with vertebral malformations, such as hemivertebrae, which were present in two French Bulldogs. Such anomalies can influence spinal biomechanics and canal geometry and therefore represent part of the real world anatomical variability of predisposed breeds. Their inclusion reflects clinical reality but may introduce additional heterogeneity; future studies may evaluate these cases separately.

Because this was a CT-based study without myelography or MRI, direct measurements of spinal cord size, compression, or intramedullary pathology were not available. Consequently, the cord-to-canal ratio, which is likely an important determinant of neurological outcome, could not be quantified. Incorporating MRI-based cord measurements in future studies would provide valuable complementary information.

Finally, canal segmentation required partial manual refinement, which introduces observer dependence, and formal inter observer variability was not assessed. Nonetheless, a standardized protocol and multiplanar verification were used to minimize error, and the magnitude of the observed between breed differences (>1,000 mm^3^) suggests that minor segmentation variability is unlikely to alter the main findings.

In summary, despite these limitations, this study demonstrates that vertebral canal volume differs significantly among predisposed breeds and correlates inversely with neurological severity in canine IVDD, supporting the potential clinical value of quantitative canal morphometry for risk stratification and prognosis.

## 6 Conclusion

Dogs of different breeds can have markedly different spinal canal capacities, which in turn influence the clinical severity of intervertebral disc herniation. In this study of Bichon Frisé, Dachshund, and French Bulldog patients, we found that French Bulldogs have a significantly larger spinal canal volume on average than the other two breeds, and that dogs with smaller canals tended to present with more severe neurologic deficits. These findings highlight canal size as an important anatomical risk factor for neurologic injury severity in canine IVDD.

## Data Availability

The original contributions presented in the study are included in the article/supplementary material, further inquiries can be directed to the corresponding author.
